# Recommended practices for computerized clinical decision support and knowledge management in community settings: a qualitative study

**DOI:** 10.1186/1472-6947-12-6

**Published:** 2012-02-14

**Authors:** Joan S Ash, Dean F Sittig, Kenneth P Guappone, Richard H Dykstra, Joshua Richardson, Adam Wright, James Carpenter, Carmit McMullen, Michael Shapiro, Arwen Bunce, Blackford Middleton

**Affiliations:** 1 Oregon Health & Science University, Portland, OR, USA; 2 University of Texas School of Biomedical Informatics, Houston, TX, USA; 3 Providence Health Systems, Portland, OR, USA; 4 Weill Cornell Medical College, New York, NY, USA; 5 Brigham and Women's Hospital, Boston, MA, USA; 6 Harvard Medical School, Boston, MA, USA; 7 Partners HealthCare, Boston, MA, USA; 8 Kaiser Permanente Center for Health Research, Portland, OR, USA

## Abstract

**Background:**

The purpose of this study was to identify recommended practices for computerized clinical decision support (CDS) development and implementation and for knowledge management (KM) processes in ambulatory clinics and community hospitals using commercial or locally developed systems in the U.S.

**Methods:**

Guided by the Multiple Perspectives Framework, the authors conducted ethnographic field studies at two community hospitals and five ambulatory clinic organizations across the U.S. Using a Rapid Assessment Process, a multidisciplinary research team: gathered preliminary assessment data; conducted on-site interviews, observations, and field surveys; analyzed data using both template and grounded methods; and developed universal themes. A panel of experts produced recommended practices.

**Results:**

The team identified ten themes related to CDS and KM. These include: 1) workflow; 2) knowledge management; 3) data as a foundation for CDS; 4) user computer interaction; 5) measurement and metrics; 6) governance; 7) translation for collaboration; 8) the meaning of CDS; 9) roles of special, essential people; and 10) communication, training, and support. Experts developed recommendations about each theme. The original Multiple Perspectives framework was modified to make explicit a new theoretical construct, that of Translational Interaction.

**Conclusions:**

These ten themes represent areas that need attention if a clinic or community hospital plans to implement and successfully utilize CDS. In addition, they have implications for workforce education, research, and national-level policy development. The Translational Interaction construct could guide future applied informatics research endeavors.

## Background

### Introduction

There is substantial evidence that computerized provider order entry (CPOE) with clinical decision support (CDS) can enhance health care quality and efficiency [1-5]. We define CDS broadly to include "passive and active referential information as well as computer-based order sets, reminders, alerts, and condition or patient-specific data displays that are accessible at the point of care [[6], p. 524]." Interest in CPOE with CDS is intensifying among clinicians and hospitals in the U.S. as federally funded financial incentives are enacted [7]. At present, only 10 to 20 percent of hospitals have CPOE [8,9], the large majority of which are academic hospitals with teaching programs or hospitals with large numbers of employed physicians, such as Veterans Affairs or Kaiser Permanente hospitals [9]. Although 86% of the 5815 hospitals in the U.S. are community hospitals [10], only 6.9% of them report having even a basic CPOE system [9]. In ambulatory settings, 17% of physicians report that they use clinical information systems, and only 4% of those physicians use systems that include CPOE and CDS [11]. The numbers, however, are rapidly rising.

Until 2006, little research about CDS had been conducted in community hospitals; nearly all had been in academic hospitals [12]. A current series of systematic reviews about the impact of CDS includes more studies from ambulatory and small hospital settings, providing evidence that the impact of CDS on patient outcomes is inconsistent, but its impact on process improvement is stronger [13-16]. A recent report notes that health information technology (HIT) is woefully inadequate in providing cognitive decision support to clinicians, other than that in patient notes and results [17]. Even worse, CPOE can actually produce numerous types of unintended adverse consequences [18], especially related to clinical workflow [19,20].

Because many problems with CDS are associated with behavioral, organizational, and cognitive issues [21,22] in addition to technical issues, the Provider Order Entry Team (POET) based at Oregon Health & Science University in Portland, Oregon conducted two multi-site ethnographic studies and convened an expert panel to focus on these issues. The first study was in community hospitals and the second in ambulatory clinics throughout the US. Their purpose was high level and broad: to identify recommended practices for CDS implementation and knowledge management. We define recommended practices to include procedures and practices actually in use at study sites (themselves exemplars) that both subjects and an external panel of experts deem worthy of consideration by other organizations. Although our main focus for this study was CDS for providers with ordering authority, we also interviewed and observed clinicians in other roles.

### Theory and framework for the study

To guide this study, we selected a systems-based theoretical framework for understanding the complexity of an organizational system such as a hospital or clinic: the Multiple Perspectives model. We have successfully adopted this approach in the past [23] to study CPOE stakeholders and describe their perspectives using qualitative methods.

**Figure 1:**
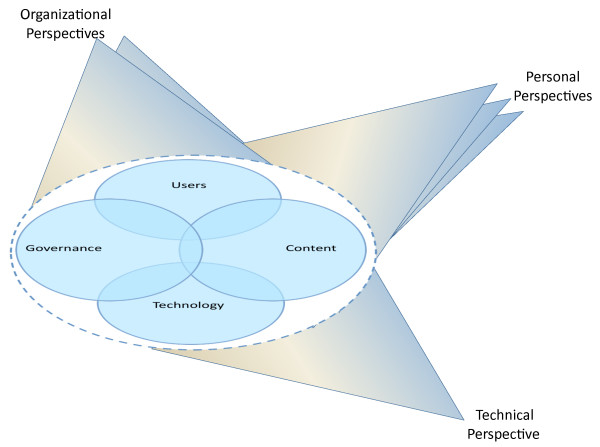
**Multiple perspectives framework for CDS study**.

The generic Multiple Perspectives model has much to offer, but to use it to structure how we approach the complexities of CDS, it needed further enhancement. The model, originally described by Linstone [24], is useful for approaching any kind of system, but it is incumbent on the model user to carefully identify the "system" (i.e., in a general systems theory sense and not as an information system). Our challenge was to break CDS into subsystems or chunks that could be explored and explained. We did this by 1) breaking the larger system (CDS) into logical components to cope with its complexity while recognizing the dynamic and nonlinear relationships among components, and 2) by using Linstone's Multiple Perspectives model [24] as a framework for studying the system. The CDS system within the dotted oval in Figure 1 contains four components we selected because they represent the major categories of issues we have identified through our grounded theory approach when analyzing field data about unintended consequences and CDS [25]. The components are user, governance, technology, and content issues. These four components overlap at times and they are all surrounded by a permeable barrier, the dotted oval, which represents the unclear boundary between the organization within which the CDS system resides and its surrounding environment. Linstone's Multiple Perspectives approach [24], (perspectives are indicated by the "wings" in Figure 1), provides a framework for how we should view the CDS "system." We need to recognize the technical, organizational, and personal aspects of what is being studied. For the technical perspective, there is only one view because it is ostensibly objective and represents one "inquiring system" [24, p. 63]. By organizational, he means the policies and procedures of the organization, as well as organizational vision, goals, politics, and culture, and there will be more than one view. By personal, he means the individual thoughts and behaviors of key players, who also hold multiple views. We used these views, or lenses through which we studied the system, to guide our subject selection, data gathering, and analysis. When we collected data from clinicians, administrators, and others, we attempted to have them see through the technical, organizational, and personal lenses as much as possible. In addition, as the researchers gathered data, they also attempted to view the CDS system through these three lenses. Finally, we selected experts for development of recommendations based upon this model. This model is a particularly appropriate framework for qualitative work because the complexity and interrelatedness of perspectives mandate a flexible yet rigorous methodology.

## Methods

A thorough description of our adaptation of the Rapid Assessment Process has been published elsewhere [26]; we will briefly review it here.

### Selection of sites

Since our goal was to identify recommended practices, we purposively selected sites with reputations for using clinical systems, including CPOE and CDS, well. We sought broad representativeness: we selected organizations with a variety of organizational structures, types of information systems and duration of use. Table 1 outlines attributes of our study sites. The hospitals were community hospitals that used two different commercial systems, one in use for over 30 years and the other for two years. Our definition of community hospitals includes non-teaching hospitals at which private physicians treat most patients. The ambulatory sites, all members of the Clinical Decision Support Consortium (CDSC) [27], used two different commercial systems and three locally-developed systems. As members of the CDSC, they were pre-selected for excellence by the CDSC Steering Committee, but they varied in maturity of information system use, type of system, and organizational structure. Many of these CDSC organizations include both academic and community components, but we deliberately sought out the latter.

**Table 1:** Attributes of study sites

	Providence Portland Medical Center	El Camino Hospital	Partners HealthCare	Wishard Memorial Hospital Clinics	Roudebush Veterans Health Administration	Mid-Valley IPA	RWJ Medical Group
Location	**Portland, OR**	**Mountain View, CA**	**Boston, MA**	**Indianapolis, IN**	**Indianapolis, IN**	**Salem, OR**	**New Brunswick, NJ**

Type of setting	**Community hospital**	**Community hospital**	**Academic and community outpatient**	**Academic and county clinics**	**VA outpatient clinics**	**Community outpatient**	**Academic outpatient**

Type of system	**Commercial**	**Commercial**	**Locally developed and commercial**	**Locally developed**	**Nationally developed**	**Commercial**	**Commercial**

Date of visit	**Dec-07**	**Feb-08**	**Jun-08**	**Sep-08**	**Sep-08**	**Dec-08**	**Feb-09**

The two community hospitals were Providence Portland Medical Center in Portland, OR, which is 1) an urban community hospital, 2) part of a larger 26-hospital system, and 3) was using a commercial system (McKesson, San Francisco, CA), and El Camino Hospital in Mountain View, CA, which is 1) an independent suburban, community hospital with the longest history of CPOE use in the world, and 2) also uses a commercial system (at that time Eclipsys, Atlanta, GA, now Allscripts). Ambulatory sites included Partners HealthCare System in the Boston, MA area. Partners' clinics primarily use the locally developed LMR (Longitudinal Medical Record) system, but a number of their affiliated clinics use commercial systems (GE Healthcare, Fairfield, CT). We studied two groups of clinics in Indianapolis, IN. Clinics affiliated with Wishard Memorial Hospital, a county hospital in Indianapolis, use the locally developed Regenstrief Medical Record System. The Roudebush Veterans Affairs Hospital, also in Indianapolis, uses the VA's nationally developed CPRS system. We also visited many clinics that are members of the Mid-Valley Independent Practice Association (MVIPA) in the Salem, OR area, which uses a commercial system (NextGen, Horsham, PA). Finally, we selected the Robert Wood Johnson (RWJ) Medical Group clinics in New Brunswick, NJ, which also use a commercial system (GE Healthcare, Fairfield, CT). We received human subjects approval from each investigator's home organization (Oregon Health & Science University, the University of Texas at Houston, Kaiser Permanente Northwest, and Brigham and Women's Hospital) and from each study site that has an Institutional Review Board (Brigham and Women's Hospital for Partners HealthCare, Providence Portland Medical Center, El Camino Hospital, the Regenstrief Institute for Wishard, Roudebush Veterans Health Administration, and the Robert Wood Johnson Medical Group), for a total of nine approvals.

### Selection of subjects

To gain multiple perspectives [24], we sought subjects who were experts in CDS content and technology and knowledgeable about CDS governance. We interviewed individuals at each site who had developed CDS, those who managed the CDS and its implementation, those who provided training and support, and users of the system. We gained additional perspectives through use of an interview field survey at the hospital sites. With the help of our sponsor and suggestions from early subjects being shadowed, we selected other users to shadow who were representative of a wide variety of clinicians, deliberately seeking out sceptics as well as regular users and champions. We continued observations and interviews until reaching saturation, the point when we were seeing and hearing the same thing repeatedly.

### Data collection methods

Researchers in the field of international health have developed expeditious methods for assessing complex site-based situations. Called the Rapid Assessment Process (RAP) [28-30], the approach uses structured assessment instruments, expert interviews, field surveys, and intensive site visits by multidisciplinary research teams. RAP is a multifaceted approach that minimizes the need for extensive fieldwork, and it has been proven to be effective [30]. RAP depends on triangulation, which is the use of multiple methods, a multidisciplinary research team, and a variety of types of settings and subjects, to gain a high level of trustworthiness in data collection and analysis.

We adapted RAP for our purposes. Before each site visit, we asked a local on-site expert to complete a "site profile," a checklist of types of CDS and questions about CDS management [31]. When possible, we also participated in an internet-based demonstration of each system so we could become familiar with local jargon and system capabilities.

Based on this information, we developed interview questions using the language of the site [26]. Topics covered during interviews were backgrounds and roles of interviewees, the culture and history of CDS, barriers and facilitators, knowledge management, governance, and the clinician view of CDS. Formal interviews were semi-structured, recorded, and transcribed. Field notes of observations were guided by the foci identified for the Multiple Perspectives Framework. Field surveys were designed to capture some quantitative data. They included short structured questions for clinicians we were unable to shadow.

Our multidisciplinary team includes clinicians, doctoral level informatics researchers with different backgrounds, and medical anthropologists. One of the most important benefits of ethnography is that ethnographers enter a culture and remain open to learning about it, thus gaining an insider view. In fact, the insiders become the teachers and the researchers become students [32]. For this particular study, the medical ethnographer on the team guided the informaticians through the RAP methodology in such a way that we became well aware of assumptions we held by virtue of our training and expertise. This attention to reflexivity was especially important during observational periods, when we had to be extremely diligent to learn about the user view and not impose our informaticians' view on activities.

After all data were analyzed and themes identified and described in writing, we convened a panel of 17 experts in May of 2010 at a retreat site outside of Portland, OR to review these results and suggest recommendations. The experts represented community hospitals, CDS content vendors and electronic health record vendors, and widely published national CDS researchers. For each theme, these experts discussed practices they would suggest for community hospitals. The format was similar to that used in a prior POET project to produce recommendations for CPOE implementation [33].

### Data analysis

In order to conduct seven site visits over two years and provide timely feedback to each site, as well as solicit comments from subjects as a form of "member checking" [34, pp. 308-9], we needed to analyze the data quickly. We did this by developing general themes during frequent debriefing sessions and using a template method [34] for roughly coding the data. For each site feedback report we identified organization-specific challenges and possible solutions. Once the site reports were completed, we began using a more traditional grounded theory approach that was both inductive and interpretive. Transcripts of the expert conference were analyzed using the template method [34].

## Results

### Introduction

We interviewed 82 subjects representing clinical, technical, and administrative disciplines. Table 2 indicates the number and roles of those interviewed and observed. We observed 105 clinicians for a total of 194 person-hours and conducted observations in most areas of the hospitals. We visited and observed clinicians working in 41 different clinics. Data analysis, which took place during 90 team meetings, revealed ten general themes.

**Table 2:** Details about interviews and observations at each site

	Providence Portland	El Camino	Partners Healthcare	Wishard	Roudebush VA	Mid-Valley IPA	RWJ	Total 7 sites
**Interviews and field surveys**								

**Roles of Subjects**								

**Admin-Managerial**	**5**	**5**	**2**	**1**	**3**	**1**	**3**	**20**

Bridger-Clinical*	**8**	**3**	**4**	**6**	**6**	**3**	**1**	**31**

**Clinical User**	**13**	**12**	**6**	**4**	**0**	**3**	**0**	**38**

**Technical**	**1**	**2**	**2**	**1**	**2**	**3**	**5**	**16**

**Site total**	**27**	**22**	**14**	**12**	**11**	**10**	**9**	**105**

**Observations**								

**Hours observing**	**36**	**26**	**37**	**20**	**25**	**33**	**17**	**194**

**Individuals** **observed**	**10**	**12**	**17**	**16**	**17**	**27**	**6**	**105**

**Number of clinics**	**NA**	**NA**	**9**	**6**	**5**	**9**	**12**	**41**

* Bridgers are generally nurses or pharmacists who bridge the gap between the clinical and technology worlds

The Multiple Perspectives model was used to help select our subjects, frame our questions and observations, and remain cognizant of relationships and dependencies among our four components of CDS: users, content, technology, and governance. It forced us as researchers to use different lenses and to gather data from users as they viewed components through the three different lenses: the technical, organizational, and personal, which also overlap and blend at times. Although the themes and patterns arose directly from our data, each is more closely aligned with one system component than others, so the four ovals depicting the CDS components within the dotted oval in the Figure 1 model will serve as an organizing scheme for the following discussion. In addition, because several of the themes that arose directly from the data did not fit into one of the four components of the CDS "system" outlined in the framework, we conducted further analysis which has resulted in our proposing a modification to the framework and a new theoretical construct.

### CDS Fieldwork Themes

Please see Table 3 for a listing of themes, subthemes, and recommended practices. Recommended practices will be described in the discussion section. Table 4 provides illustrative quotes for each of the themes.

**Table 3:** Themes, subthemes, and recommended practices

Theme	Subtheme	Recommended Practice
**User Component-- Theme 1: Workflow**		

		Assess workflow early

		Start with simple inline CDS

		Plan to customize products to fit workflow and vice versa

**Content Component-- Theme 2: Knowledge Management**		

	Knowledge creation	Plan early and allocate sufficient resources

	Content library management	Catalog and monitor CDS from the beginning

**Technology Component-- Theme 3: Data as a foundation**		

	Having enough information about the patient	Develop interfaces to external systems

	Quality of the data	Participate in HIE efforts

	Sharing the data	Educate clinicians about reason and importance of good data

	Varied uses of data	Promote standards

**Technology Component-- Theme 4: User computer interaction**		

	Customization	Solicit feedback

	Usefulness	Test new CDS on users

**Technology Component-- Theme 5: Measurement and metrics**		

	Administrative needs	Identify reporting goals

	Monitoring CDS	Plan measures early

		Refine CDS based on measures

**Governance Component-- Theme 6: Governance**		

	Environmental factors	Identify existing structures to repurpose as many as possible

	Setting priorities and resource management	Establish decision making structures early

	Governance structure	Plan to reassess structures regularly

	Relations with vendors	Involve clinicians continuously

**New Construct, Translational Interaction--Theme 7: Translation for collaboration**		

	Collaboration for development	Promote collaboration

	Translation for vendor collaboration	Speak language of collaborators

	Translation between users and IT	Spend time with users as they work

	Collaboration among clinical orgs.	Create a culture of interaction

**New Construct, Translational Interaction--Theme 8: The meaning of CDS**		

	Multiple meanings	Understand the user view

	Informatics philosophy	View CDS broadly

**New Construct, Translational Interaction--Theme 9: Roles of special essential people**		

	As previously defined	Create and formalize roles

	Newly found roles	Educate the workforce

**New Construct, Translational Interaction--Theme 10: Communication, training, support**		

	Communication, training, support	Involve users, give resources, over-communicate

**Table 4:** Representative quotes from the fieldwork

Theme 1: Workflow	Theme 6: Governance
"People practice in very different ways. Some physicians look at the screen once before they see the patients, and then they don't really touch the computer [again] until they have to write prescriptions. So, the opportunities to interact with the computer and receive decision support can be limited for those practitioners." "Now they need to turn the alerts back on, condition by condition. They plan on customizing the alerts before they turn them back on." "She uses the point and click charting templates to complete her review of systems [and] history and physical very quickly."	"We moved to the EMR because we felt it would standardize or help quality." "I think the underlying drive, perhaps not surprisingly, it's the recognition that we need to distinguish ourselves as an organization from amongst the competitors." "We sort of have a very tight knit connection with [our vendor]. So, I think everyone sort of collaborates with them and cross-communicates with them on practically everything."

**Theme 2: Knowledge management** "We assess new drugs as they are introduced as possible candidates for CDS."	**Theme 7: Translation for collaboration** "She is a 'development analyst' and the team leader for similar analysts. They write specs, test, and modify and they serve as liaisons between the users and IT. There are other analysts who are implementation and support analysts." "A lot of her job and a lot of my job is working with [the vendor] to make sure things are running correctly."

**Theme 3: Data as a foundation for CDS** "The patient we were looking at had an LDL reminder but the patient had actually had the LDL done. The reminder didn't work correctly since he didn't have lab results in the EMR (so it thought the test hadn't been done when it had been)."	**Theme 8: The meaning of CDS** "Sometimes the best decision support is not to give them [physicians] the decision [and to design a nursing protocol instead]."

**Theme 4: User computer interaction** "We have to build custom-like orders, we have to build for the practice, medications, problems, custom lists for each practice and when they log into the system, it automatically defaults to their custom list." "It flags it in red, so it's a visual cue to the physicians that it's a little bit outside of the range and if you're ordering something with a narrow therapeutic index, you need to be aware."	**Theme 9: Roles of special essential people** "We do have people who are practicing clinicians who are helping create the rules. You definitely need someone who knows the technical side of the equation." "I know how challenging it is for clinicians to take time to address these important issues. That has been compensated."

**Theme 5: Measurement and metrics** "Most physicians will use the reminders because they get report cards on their completion rates. . . if you go down into the clinics you'll see graphs that compare clinics to one another as a form of competition." "We are now better able to track the timeliness and the labor required to meet those maintenance obligations."	**Theme 10: Communication, training, support** "There are always new features that come up and I think we still completely suck at letting people know about these new features." "We actually make it very easy [for clinicians] to write patients a letter describing their test results in a patient-friendly format." "We have to understand what the physician is going to be doing. Are they going to be dictating, typing their notes? So, really trying to gear the training around workflow."

### Component One: Users as a component of the CDS "system"

The end users of CDS are those whose workflow is most affected by it. Users are constantly adjusting their work because of the system and the systems are ideally constantly changing to better facilitate users' work.

### Theme 1: Workflow

We were consistently told that any system should fit the workflow of its users as closely as possible. The locally developed systems were designed to fit into the work done at a particular site, but since users differ in their work habits, even these systems needed some customization to match individual workflows. Concomitantly, users must generally adapt their workflows to better fit the system. Those using commercial systems are continuously individualizing or customizing aspects of the system to better fit their ways of doing things, or adapting to the system's requirements. There are limits to what buyers of commercial systems are allowed to customize, however, which is often why workflow must be adapted.

### Reengineering the workflow

The sites using commercial systems had all conducted workflow analyses in each clinic prior to implementation. The sites with locally developed systems seem to be in a perpetual state of workflow engineering. A researcher wrote in fieldnotes: "I speak to the workflow fellow who calls himself an EMR Workflow Engineer. He observes how the staff uses the computer system and helps them to trouble shoot workflow problems. His team observes the lean manufacturing/production philosophy. He uses time/motion studies and asks the practice about what needs they have." At one site we were told "So now what we're doing is we're sort of going back to all these sites and saying okay, we're going to start from scratch with you. We'll go over all of your workflows and all the ways that you document and make your decisions and we'll show you how to do this in the EMR now."

### In-line applications and CDS that fit the workflow

By in-line applications we mean computer-enabled help that seamlessly fits the workflow, that does not interrupt the clinician, and that is nearly invisible. Applications are in-line if they provide needed information at the appropriate time in the encounter. Templates are an excellent example of an in-line application providing decision support. These were especially useful in the ambulatory setting when clinicians used the system during the patient encounter. One researcher's fieldnotes said: "She uses the point and click charting templates to complete her review of systems [and] history and physical very quickly." Another noted: "[The provider] uses templates and occasionally brings notes forward. I asked him whether he did this because it made it faster or because it helped him remember. He said mainly because it made it faster, occasionally for remembering." Some users were critical of the documentation generated by use of the templates, so they entered free text into the template instead of or in addition to filling in the fields. Some clinicians would not use a computer in the exam room because they thought it would hamper physician-patient interactions. Some, however, were observed to be remarkably facile, brought the patient into the encounter skilfully, and enjoyed using the templates. Often, these were the clinicians who had taken the time to modify templates to their liking.

Most clinicians who had e-prescribing available praised its ability to help them. One researcher said in fieldnotes: "If he prescribes a med, he does it in the room on the computer. It [then] prints out [so he can] hand it to the patient or to fax, or may fax directly. The app is populated by a list of pharmacies. The patient's usual choice is there as the default value."

### Variability of workflow

We were told that the prime reason why workflow analysis is needed prior to implementation or on an ongoing basis is that each physician has developed his or her own way of doing things. One interviewee said: "People practice in very different ways. Some physicians look at the screen once before they see the patients, and then they don't really touch the computer [again] until they have to write prescriptions. So, the opportunities to interact with the computer and receive decision support can be limited for those practitioners." Others carry laptops or tablet computers with them at all times and have multiple opportunities to receive CDS.

### Location of the encounter

We observed that CDS usefulness depends a great deal on where the physician opts to use the computer. Clinicians who use templates during the patient encounter receive timely, helpful, welcome, seamless decision support: "Her process is to use her laptop in the exam room, filling out the smart form [template] for her note. She further edits the smart form in her office." On the other hand, clinicians who waited to use the templates, often until after the patient had left, missed an opportunity to be reminded of important issues.

### Temporal issues

Timing of the CDS presentation, especially alerts, is important to users. We heard complaints about alerts firing at the wrong time, both too early in the encounter and too late. Clinicians wanted them at "the point in time during the encounter where it's really going to be most helpful and most actionable." Time pressures had an impact as well. None of the outpatient sites we studied had many alerts aimed at physicians. We were told: "They're overwhelmed, they're too busy, they have too many demands on their time." An informatician noted about alerts: "We thought that it was a much bigger downside to frustrating people by constantly interrupting their workflow than missing the alerts." One site turned them all off and a representative told us: "Now they need to turn the alerts back on, condition by condition. They plan on customizing the alerts before they turn them back on; having the task force review the logic before they turn them back on; turning them on clinic by clinic."

### Component Two: Content as a component of the CDS "system"

Content issues include development or purchase and management of CDS.

### Theme 2: Knowledge Management

By knowledge management, we mean the entire process of developing and translating pieces of knowledge so that they are available in the system. Knowledge management also includes acquiring, tracking, evaluating, and maintaining knowledge, just as libraries gather, catalog, and maintain library collections.

### Knowledge creation

The sites we studied that had locally developed systems also had locally developed CDS, which, because of the way it was developed over many years by innovative individuals, is hard to track. However, they are making progress in developing ways to monitor their CDS. Individuals at these sites continue to develop new CDS, which now tends to be more carefully managed. Close ties with the pharmacy and therapeutics committees and quality assurance staff members yield ideas about "new drugs as they are introduced as possible candidates for CDS." Although our study sites that used commercial systems did not develop CDS de novo, they followed many of the same processes when customizing content they obtained from others, including content vendors. All of our sites with commercial systems had informaticians in leadership roles and CDS analysts who could modify CDS content.

### Content library management

We use this term because we see an analogy between traditional library functions such as acquisitions, cataloging, maintenance, provision of access, and "weeding" of materials and the functions that appear to be needed for CDS content management. The acquisition phase includes either development or modification of CDS, described above. Once an organization acquires a certain amount of CDS, it starts to lose track of what it has, so an inventory is wise if it has not been conducted from the beginning. Following the inventory, a means of cataloging, or indexing, is needed so that analysts can search to find out what exists. Model sites conduct cyclical reviews for curation and maintenance and have mechanisms for scanning the environment to keep up to date about new evidence. Organizations with commercial systems can take advantage of software offered by vendors to help manage this process. Unlike libraries, holders of CDS do not often share their locally developed or modified CDS. We asked interviewees about their willingness to share CDS. The reasons for not sharing included lack of a technical ability to do so and a hesitation to share without remuneration what they had spent time developing. There are also legal issues that inhibit sharing. On the other hand, there was interest in sharing among sites that have a particular vendor-based system and also when an organization wants new CDS. Organizations would like to be on the receiving end but not the giving end of the exchange.

### Component Three: Technology as a component of the CDS "system"

Several of our themes relate to this component: data as a foundation for CDS; user computer interaction; and measurement and metrics.

### Theme 3: Data as a foundation for CDS

Many types of CDS require that data about individual patients reside in the system. For example, before a reminder that a mammogram should be scheduled can be generated, the system needs to know the age and gender of the patient, when her last mammogram was performed, whether they have a mammogram already scheduled, and finally, if they are status post bilateral mastectomy or in a hospice program. We were told that if decision support is to be highly sensitive and patient-specific, then accurate, complete, structured information about the patient must already exist in the system.

### Having enough information about the patient

None of our study sites has truly complete data about its patients because patients receive care from many different organizations. Even VA patients sometimes get care outside of the VA system. Some data, such as those in medication lists, are especially hard to keep accurate and up to date. Other data must come from sources such as laboratories and agreements as well as technical interoperability are needed if these data are to be shared. For example, one interviewee noted: "The patient we were looking at had an LDL reminder but the patient had actually had the LDL done. The reminder didn't work correctly since he didn't have lab results in the EMR (so it thought the test hadn't been done when it had been)." We often heard remarks such as: "We're working on getting university radiology, a radiology site to send us their results electronically, because right now they come over as paper and we have to scan them." Clinicians uniformly desire having all the right information, but not too much information, at the point of care.

### Quality

Often our subjects worried about the accuracy of data that had to be entered manually. One said "Even my own partner doesn't really, you know, capture or do the data. I mean a lot of it is just getting the work done at that moment in time." A nurse confided that "at times the nurses will simply cut and paste medication profile information from [the system] into the medication reconciliation document without properly verifying all of the medications on the list."

### Sharing Data

Data are often stored in separate silos, with laboratory and radiology information in separate systems that cannot share information with an EMR. The extent of the ability to share data within and across sites varies a good deal. One site, which included a group of outpatient clinics, shares nothing but demographic data between clinics. The VA shares nationally, Partners sites share some information such as allergy information, and the Wishard clinics using the Regenstrief system are part of a statewide network sharing some patient-specific clinical information.

### Varied uses for these data

Administrators and informaticians told us they value data availability not only as a basis for patient specific CDS but also for quality measures reported after the fact. One informant said "It is frustrating that we have not been able to get any quality indicators out" because data were not being entered by all clinicians. Another use is for research purposes, and both accurate and complete data are needed. Others would like population-based data.

### Theme 4: User computer interaction

We think of user computer interaction as ease of use of the system, including the equipment, the screen layout, the number of clicks needed to accomplish a task, the cognitive energy needed to figure out what to do next, and the speed of use. There are two major sub-themes that emerged: customization, which can take place on many different levels, and usefulness.

### Customization

All of the systems we studied could be customized and these successful sites all devoted considerable staff time to this endeavor. Even sites with locally developed systems were constantly providing further customization: "We sat down with the [EMR] team and they had to change the user interface of how pediatricians would order medications, because now we're doing it through weight based dosing vs. flat dosing." An analyst at a site with an EMR that provides templates noted: "We can edit the forms and customize them for the practice. We have to build custom-like orders, we have to build for the practice, medications, problems, custom lists for each practice and when they log into the system, it automatically defaults to their custom list." Analysts also make changes to simplify use of the system. As one analyst noted: "I look at it and say no, this will never fly. Ten clicks to get here, forget it, we've got to simplify this."

### Usefulness

During observations, we found that presentation of the CDS was of utmost importance. Where CDS was "in-line" with workflow, we often observed that simple presentation decisions could be extremely powerful. For example, the use of color draws attention to data without changing workflow: "It flags it in red, so it's a visual cue to the physicians that it's a little bit outside of the range and if you're ordering something with a narrow therapeutic index, you need to be aware." Actionability was likewise critical. If reminders are "actionable," meaning that the clinician can respond to the reminder without needing to access another part of the system, usually with one click, they minimize impact on workflow and tend to be used. At one site, most decision support is provided this way, and the positive outcome is that reminders tend to be voluntarily viewed and acted on. In addition, structured data are collected and reports generated about responses to reminders. Reliability is very much valued by clinicians. We were told that CDS cannot be useful if clinicians avoid the system because it is not available at times. Finally, correctness and applicability to the patient are important. There are times when the system simply is not correct. One clinician noted: "When you order inhalers, it often rejects the dose that it suggested you use!"

### Theme 5: Measurement and metrics

We were told that patient-specific, accurate, and complete information that already exists in the system is needed to measure both the effect of clinical decision support and the use of it. Also, metrics need to be established so that the impact of the EMR can be measured over time. For example, it is useful to know how often alerts are being overridden and why.

### Administrative needs; quality reporting

With increasing pressure to be accountable for quality, the sites we studied either already take advantage of measures that can be extracted from the system based on CDS interventions, or they are planning to conduct ongoing measurement once the system is fully implemented across all clinics. We were told at several sites that provide performance feedback to clinicians that such feedback is welcomed. One site with a "dashboard" provides direct feedback to clinicians. An interviewee noted: "So, it's at the clinician level, sort of their performance on key indicators compared to their peers and compared to some external benchmarks." Some sites provide incentives for meeting performance goals: "we are reporting on things that ultimately become these accountability metrics. . . people are either going to get bonuses if they do certain things and if they don't, they don't." At another site we were told: "most physicians will use the reminders because they get report cards on their completion rates. . . they are attended to as opposed to other systems in other organizations where there's not this tracking reporting type system. If you go down into the clinics you'll see graphs that compare clinics to one another as a form of competition."

### Monitoring and control of CDS

Some of the study sites monitor how effective some CDS is: "How usable is our decision support such that for example we are now putting in routine efforts to track override rates." They might also monitor the effort put into maintaining CDS: "we are now better able track the timeliness and the labor required to meet those maintenance obligations." Sites that do not monitor CDS at present are planning to do it soon.

### Component four: Governance as a component of the CDS "system"

All of our study sites had formal governance structures for managing CDS.

### Theme 6: Governance

Governance includes formal and informal mechanisms for making decisions about the system and about CDS in particular; four subthemes emerged from our data.

### Environmental factors/Motivation

We were told in interviews that while the ultimate motivator for implementation of CDS is the desire to improve patient care, there are other intermediary factors pressing for it. These include increasing attention to rewarding patient safety and healthcare quality by accrediting bodies, payors, and professional societies. As one quality assurance director noted: "We moved to the EMR because we felt it would standardize or help quality and would standardize our, some of our practice." Another motivator is competition in the health care sector. As one interviewee stated: "I think the underlying drive, perhaps not surprisingly, it's the recognition that we need to distinguish ourselves as an organization from amongst the competitors in terms of safety and quality."

### Setting priorities and resource management

We were told that one of the most difficult aspects of CDS governance is the setting of priorities. With outside pressures to meet certain measures and internal pressures to decrease costs or improve specific local outcomes, organizations must decide where to put their energy. One of our sites has a committee that developed a list: "A top ten list of what we thought should be standardized across the enterprise." Each site has a somewhat different approach to setting priorities, but all have multidisciplinary committees that provide oversight and make ultimate decisions.

### Governance structure

We found three aspects of governance structure related to CDS, which our study sites consider crucial: committees, process, and feedback to the governance system. Committees play a vital role in governance. The more mature CDS sites have several layers of committees, with higher-level decisions made by higher-level committees. These are generally multidisciplinary, with a mix of clinicians, administrators, and technology representatives. One was described by an interviewee: "It's called the EMR IT Advisory Group. The physicians, some of the IT staff, some of the clinical staff, and the analysts." All tend to gather task forces of clinical experts when needed. Each site has a process for discovering new evidence or environmental changes that impact CDS. One example of this is a process for learning about changes made by the Pharmacy and Therapeutics Committee. Finally, mechanisms for feedback to the governance system are imperative. Each site also has a process for reviewing requests from users. At one site, "the clinical content committee reviews requests that come up from the user base and they are funnelled to and from the [information system] management team about decision support."

### Relationships with vendors

The sites with commercial systems must depend a great deal on decisions about CDS that are made by their EMR vendors. Therefore, they must be in close contact with the vendor. We heard at one of these sites: "We sort of have a very tight knit connection with [our vendor]. So, I think everyone sort of collaborates with them and cross-communicates with them on practically everything. We really can't do very much on our own here without [the vendor]." The EMR vendors generally purchase content for CDS from content vendors. Sites with locally developed EMRs often purchase directly from content vendors, especially for medication information.

### Proposed New Construct, Translational Interaction

Several themes emerged from our analysis of the data that did not easily fit into the framework originally proposed, which included components for content, users, governance, and technology. Instead, the additional themes all included aspects of "translation," which we define as communicating meaning through language.

### Theme 7: Translation for Collaboration

For groups to collaborate effectively, they must understand the cultures of the different involved groups. Culture implies a shared system of meaning and language. The different groups for which collaboration is necessary include: the developers and analysts, IT staff, clinic staff, the vendors, clinicians, and administration.

### Collaboration for development

At sites that build new CDS, the development process involves a development analyst who facilitates discussions among clinical specialists, knowledge engineers, and programmers. A researcher explained in fieldnotes: "She is a 'development analyst' and the team leader for similar analysts. They write specs, test, and modify and they serve as liaisons between the users and IT. There are other analysts who are implementation and support analysts." The development analysts and knowledge engineers exist at the interface of the clinical and information technology worlds and are familiar with the vocabularies of both.

### Translation for vendor collaboration

At the sites with commercial systems, analysts modify CDS content provided by vendors and to do so, they must often work with members of the vendor's staff. As one analyst stated it: "A lot of her job and a lot of my job is working with [the vendor] to make sure things are running correctly." Often, those within the purchasing organization feel that they are not sufficiently supported by vendors, although it is usually incumbent on them to purchase services in order to receive them.

### Translation between users and IT

Both analysts and training and support staff translate or explain the clinical culture to information technology staff. One analyst noted: "Our local IT people said 'oh, that can't be done. . . then I realized it wasn't that it couldn't be done. The IT people we were working with didn't understand what the clinicians were asking to get done. I realized then that there needs to be some kind of an intermediary who understands the IT world and the clinical side."

### Collaboration among clinical organizations

The cultures of outpatient clinics which house physicians in private practice and the cultures of the hospitals to which those physicians refer patients are different enough that information systems are impacted. The business models are different, of course, and some vendors do not have products for both or will not sell to both except under certain circumstances. In addition, there is sometimes competition for patients if the hospital performs outpatient procedures. We were told that collaboration between the organizations that purchase content and EMRs and the vendors is essential.

### Theme 8: The Meaning of CDS

We asked each interviewee how he or she would define CDS, primarily because we wanted insight into different perspectives. We quickly learned that CDS generally means something quite different to the informatics experts than it does to the clinical users. It is important to note that meaning goes beyond definition: it is making sense of a phenomenon. Much of what we learned about these mental models our subjects hold came from observations and informal comments. Their view surprised us: often they did not know what "clinical decision support" is, so we had to explore the idea by asking how the computer helps them make clinical decisions. The users see CDS as an opportunity for the system to help them get through their day. They focus on the help and assistance the EMR can offer. Experts usually describe CDS in terms of sophisticated alerts or reminders. Interestingly, CDS implementers at each site seem to have a unique philosophy that guides their CDS efforts, a shared mental model or organizational meaning. We divide this theme into two sub-themes: the multiple meanings of CDS and different informatics philosophies of CDS.

### Multiple meanings of CDS

Clinical decision support means different things to different disciplines and to individuals within those disciplines. One of our sites has a position called "Coordinator of Clinical Decision Support" which deals exclusively with administrative data in the form of reports about clinicians' actions and not at all with how clinicians make clinical decisions. We heard similar definitions echoed by quality assurance staff. When asked for a definition, informaticians usually offered a very broad definition such as "presenting information to somebody in a way that's going to help them to make decisions or take actions." However, those individuals often went on to describe alerts and reminders, most likely because these forms of CDS are most interesting to them. On the other hand, most of the practicing clinicians we observed thought of CDS as anything that could help them finish their work in a timely manner. Any information in either the clinical information system or the office practice system that assists the clinician's workflow constitutes CDS in their view. Some clinicians described talking with or e-mailing other clinicians or even reading another clinician's notes as decision support. They make little distinction between clinical information and other types of information such as demographic or scheduling information. To them, all of this information helps them take care of their patients.

### Informatics philosophy of CDS

Experts at several of our sites expressed philosophies that guided their organization's development of CDS. One of the study sites held a philosophy "we're not trying to tell the physicians what to do, we're trying to give them the information." Informants at another site used the terms "guardrails" and "helping the clinician to do the right thing." One informatics professional noted: "I've seen a lot of decision support done as forcing people down this path or that path, always has been the carrot or the stick. . . what is the grade of the ground? You can get that mule pulling that cart, are you whipping them, are you enticing them, but the truth is that if you just make it easier to go down one path. . . To me that's the ideal decision support is when the person doesn't even realize that it is happening." Elsewhere we heard "giving vaccinations when patients were in the hospital and the most effective way to do it was to give it to nursing and make it part of their protocol; and take it out of the decision tree of the doctor. Sometimes the best decision support is not to give them the decision." Administrators at one site clearly saw CDS as an "enabler of standardization."

### Theme 9: Roles of Special, Essential People for CDS

In prior studies, we have identified and described typical "special essential people" roles for CPOE implementation and maintenance [35]. These roles include administrative leaders, clinical leaders, champions, opinion leaders, and bridgers of different types who span the gap between the clinical world and the technology world and generally provide support and training. This study confirmed that these roles are critical for effective CDS as well. However, this study also identified several new and emerging roles directly related to CDS, which will become increasingly important. We arrived at this list through analysis of statements our informants made during interviews and through field observations.

### Essential people as previously defined

We found the same types of essential people at these sites that we described after visiting five organizations for a prior study about CPOE success factors [35]: champions, who are clinicians in the forefront of information technology; opinion leaders, who are clinicians well respected for their clinical expertise who are spokespeople for systems; administrative leaders, who are not clinicians, but who hold a vision of what CIS can do; clinical leaders, who are clinicians by background but hold administrative positions; and "bridgers," who are usually clinically trained but who have enough IT expertise so they can train and support users or serve as analysts who develop or modify systems. These designations are not mutually exclusive, since clinical champions may also be administrators, for example. In addition to these roles, we discovered in this CDS study a number of variations of the roles we previously identified. For example, two sites that have commercial systems have on-site analysts who actually work for the vendor and not for the hospital or practice. This arrangement has both advantages and disadvantages and seems to work best when the analysts have experience working for the organization they serve. One analyst of this type described how difficult it had been for outsiders hired as analysts because "They had to get used to the flow. . . they had to get used to that because this is a completely different environment for them. So it took them a while to kind of figure that out, whereas I already knew, that so that was an advantage for me."

### Newly found essential roles for CDS

These include knowledge engineers, subject matter experts, outpatient clinic champions, pharmacy informaticians, and ambulatory clinic chief medical information officers.

#### Knowledge engineers and analysts

The sites we studied that have non-commercial systems were unique in that they each have knowledge engineers who are clinicians, usually physicians, who have developed and evaluated decision support through grant funding. These knowledge engineers help to develop the content for CDS and are skilled facilitators who seek to gain consensus from clinicians. They translate human readable content into a form that the system can use, so they are technically as well as clinically astute. Their role was well described by one informant: "We do have people who are practicing clinicians who are helping create the rules. You definitely need someone who knows the technical side of the equation." Analysts are generally clinically trained as well and they perform many of the same tasks of knowledge engineers, often modifying content available through commercial systems.

#### Clinical CDS Subject Matter Experts (SMEs)

Each organization has a cadre of clinicians who assist with development or modification of decision support. Organizations seem to have difficulty motivating SMEs as time goes on. By SME motivation, we mean that once these individuals are identified and they are taking their SME roles seriously, they need to be nurtured and continuously updated and motivated to continue being SMEs. These people are clinicians who are interested in information technology and the potential of computerized CDS. When systems are new, they seem naturally motivated by the challenge of implementation. However, as CDS is continuously rolled out, these SMEs grow weary. Some of the sites compensate these experts: "I know how challenging it is for clinicians to take time to address these important issues. That has been compensated and I think that's another whole, you know, another whole dimension."

#### Outpatient clinic champions, often non-clinical

In office practice settings, there is usually someone who serves as office champion. This go-to person is sometimes one of the clinicians who has an aptitude for computer systems, but more often it is one of the office administrative staff members who devotes part time to working with the system. Having such a point person who is both knowledgeable and personable seems to be important for success.

#### Pharmacists who are Pharmacy and Therapeutics (P and T) Committee connectors

Hospitals have Pharmacy and Therapeutics committees, which oversee medication use. Pharmacists who bridge the gap between these P and T committees and CDS developers are uniquely capable of assisting with medication related CDS development and maintenance. They play a critical role in communicating between the committee and the developers so that the developers are well informed about P and T priorities.

#### Ambulatory clinic CMIOs

Each of the two freestanding clinic organizations we studied hired physicians with informatics training to fill the role of a chief medical information or information systems officer. Each used a commercial system, so these individuals played a key role in expressing the clinic's needs to the vendor, in facilitating the work of the CDS analysts, and in communicating with users.

### Theme 10: Communication, Training and Support

Communication, training, and support are critical success factors for any clinical information system, but there are unique issues when the focus is CDS. This theme includes communication and training about new CDS and CDS modifications as well as ongoing support efforts.

Communication about CDS takes many forms, and staff members at our study sites feel it is exceedingly difficult. Like training, communication is hard to do when busy clinicians are the target audience. One informatician said: "there are always new features that come up and I think we still completely suck at letting people know about new features." Types of communication include e-mail, on site meetings and presentations, "lunch and learns," use of feedback buttons, and personal contacts with CDS analysts or super users. Communication between clinicians and patients, we found, can be enhanced through CDS, both through the use of reminders about health maintenance schedules and other forms of communication. For example, one informatician noted: "We actually make it very easy [for clinicians] to write patients a letter describing their test results in a patient-friendly format."

Training, primarily conducted one-on-one at the sites we studied, needs to be ongoing, especially as more decision support is added, and organizations find it exceedingly difficult. It is hard to separate training from communication and support or to differentiate it from education or efforts to motivate clinicians. As one physician developer noted: "asking them [providers] to do something with decision support, it's just, you know, to really make that behavior change requires lining up more than the reminder. You've got to line up education and incentives and a whole bunch of things and generally we don't do that for too many [providers]."

Initial training needs to show the user how a particular CDS type might fit that user's individual workflow. One trainer noted: "We have to understand what the physician is going to be doing. Are they going to be dictating, are they going to be typing their notes?. . . So, really trying to gear the training around workflow." Ongoing training is especially necessary as new CDS is added. However, organizations were somewhat apologetic about their inability to do this well. We found that users rarely felt their knowledge about changes to CDS was up to date. One physician said "A lot of it you learn by trial and error," and a researcher's fieldnotes noted someone "had developed some work-arounds that seemed valuable, but that required him to do many inefficient actions within [the system]."

By support, we mean providing help to users at the time of need. Support related to CDS involves continuous feedback to and from users, generally by phone to a help desk or through e-mail. One clinician's quote is characteristic of what we heard at all of our study sites: "whenever we e-mail them [IT support] or have a problem with them or feel like things should come up differently or pop up differently or whatever, I mean, they're great."

## Discussion

### The themes

Many of our results, including the importance of workflow integration, well designed user interfaces, ongoing knowledge management and intentional interaction among stakeholders, confirm statements made by others based on their experience and expert opinion [6,36-41]. While much of what we have described may seem familiar, one characteristic of good qualitative research is the ability of those studied to see themselves, and their world, reflected in the results. In grounding the findings in carefully collected data from seven varied sites, this research validates and strengthens previous work. It also extends the findings to community hospitals and ambulatory clinics, sites which are historically under-represented. In addition, we offer actionable recommendations based on our findings, thus furthering the ability of hospitals and clinics to increase the quality, safety and efficiency benefits from CPOE with CDS. The process for identifying recommended practices was as follows. First, interviewees were asked about them. Second, observers recorded noteworthy practices in fieldnotes. Third, debriefings and team analysis meetings identified them for the reports. Recipients of the site reports were asked for feedback as a form of member checking. Finally, descriptions of recommended practices were presented to the panel of experts during a two-day conference and discussed at length. Those recommendations are offered below and included in Table 3.

### Recommendations about users as a component of the CDS "system"

#### Theme 1 Workflow

Organizations should pay attention to workflow assessment prior to any intervention. There are good examples of CDS types that fit the workflow of users, with order sets and templates among the best. Even the smallest clinics we visited were using these with success once some customization had been done. These are good places to start, and any organization with an EMR can do it. Higher level, interruptive CDS types like alerts need careful screening and they should be a goal, carefully planned with clinician involvement. We recommend that organizations with EMRs move forward with simple CDS no matter what their size. Order sets, checklists and templates can be considered "low hanging fruit" and they suit the purposes of all stakeholder groups by providing standardization and gathering structured data that can serve multiple purposes. Some of this low hanging fruit is available from vendors, but, as others have noted in the past, organizations must plan on customizing it so that it fits local practice and workflows [20,42].

### Recommendations about content as a Component of the CDS "system"

#### Theme 2 Knowledge Management

Organizations should plan early and establish procedures for the maintenance of CDS. Knowledge used in CDS changes rapidly, so organizations should have the resources to purchase and to keep knowledge bases up to date. Some organizations avail themselves of services offered by content vendors to help them to manage their CDS. At a national level, if there is greater utilization of Continuity of Care Document (CCD) standards, making it easier for external applications to individual patient data as part of the clinical workflow, "clinical content development organizations will begin to make available actionable, real-time, clinical decision support interventions on a widespread scale. [[43], p. 616]"

### Recommendations about technology as a component of the CDS "system"

#### Theme 3 Data as a Foundation for CDS

Organizations should take all possible steps to assure acquisition of high quality data. Clinicians should be educated about why good structured data are needed and why data integrity is so important [44]. At the national level, standards for health information exchange are evolving, and they should be supported. Standard triggers, which cause certain decision rules to be invoked, need to be defined. Finally, standards for input data need to be defined. Of course, use of standard vocabularies is needed so that CDS can be both robust and shared across implementations.

#### Theme 4 User Computer Interaction

As CDS is developed, it should be tested on real users prior to implementation. Mechanisms need to be in place for receiving user feedback and acting on it [40]. The in-line CDS described here is most usable in that it does not interrupt workflow. If CDS must interrupt workflow, it should be designed so that it is actionable. In other words, the user should be offered choices that can be selected immediately, without navigating to another part of the system.

#### Theme 5 Measurement and Metrics

Measurement and refinement of CDS content is critical for CDS interventions to be effective. Organizations should design metrics as content is developed or purchased, and they should be diligent about implementing the measures. Once measurements are available, reports should be communicated and the CDS interventions should be refined as needed. Decision makers need to plan what will be measured as early as possible, and each stakeholder group should be included in the decision-making. Clinicians themselves will be interested in measures of their own clinical patterns, implementers will be interested in how well the CDS is working, and administrators and quality assurance staff will desire measures of safety such as those required by accrediting bodies.

### Recommendations about governance as a component of the CDS "system"

#### Theme 6 Governance

CDS is a powerful tool for influencing clinician behavior. It is important to have an effective governance process in place to keep clinical leadership, end users, and IT aligned. Existing committees like a Pharmacy and Therapeutics or quality committee may be able to serve this purpose, but in many cases, they will need to be modified or new committees will have to be developed, especially as the content grows and becomes more complex. Clinicians must be involved and, to motivate continued involvement, a suitable reward system is needed.

#### A new theoretical construct: Translational interaction

We crafted our questions and foci for observations around the four components of users, governance, technology, and content, so it was not surprising when the themes of workflow, governance, usability, measures and metrics, data as a foundation for CDS, and knowledge management were identified as themes. However, four themes spontaneously and surprisingly arose from the data: translation for collaboration, the meaning of CDS, new roles for essential people, and communication, training, and support. When we realized that they all had in common the notion that meaningful exchanges between actors with diverse worldviews are difficult but critical at the points of overlap that exist among our original four components, we sought further insight about the commonalities of these four themes.

The medical anthropologists on the team were familiar with the work of Michael Agar, which seems especially applicable here. He describes Rich Points, which occur at the point of interaction between actors with different understandings of a situation and lead to "moments of incomprehension and unmet expectations [45]." According to Agar, Rich Points like these require a *translation *between the different ways of understanding, or worldviews, in order to explain the meaning of the situation. Translation is especially difficult because it goes beyond words and vocabulary and includes cultural meaning. For example, "system" to a physician might mean the physiological system but to an information technology staff member it usually means hardware and software. In fact, Agar has coined the term "languaculture" to emphasize that language and culture shape one another. Informaticians, for example, often bridge both worlds and can explain the cultural and language differences between them. We adopted the term "translation" from Agar because the languages of the clinicians and the information technology workers are different and the languages need to be mutually understood by individuals involved in CDS. "Language is not a prison," according to Agar, however: "it is a room you are comfortable in--you can move out of it but it is uncomfortable [[46], p. 68]." Successful negotiation of these Rich Points leads to shared understanding and expectations, which in turn enables communication and action. Our team noted that translation alone, with its focus on language and culture, fails to take into account this process, which includes moving among worlds. For example, a physician may collaborate with others working to modify a CDS module by virtue of his clinical expertise, but he may hold a meaning of CDS as "alerts and reminders" by virtue of informatics training. That same person might fill the role of a new kind of essential person as a knowledge engineer, and may spend part of his time training other physicians as their peer. In other words, there is active movement between and among the components, so we are calling this aspect Interaction. The entire process of building this shared system of meaning and language (or, integrating multiple systems of meaning and language) across disciplines and worldviews we call Translational Interaction.

**Figure 2:**
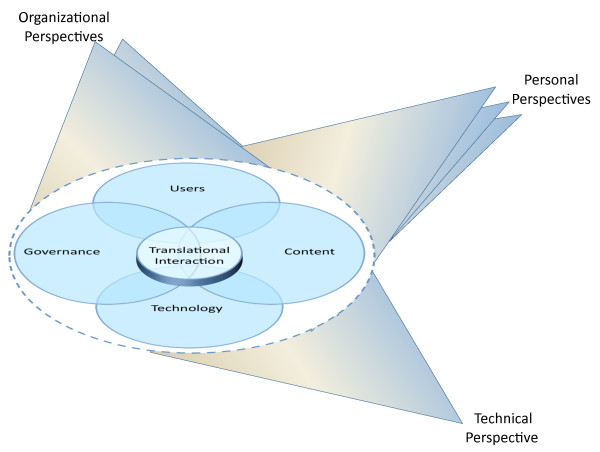
**Revised Multiple Perspectives Model**.

It seems to us that the intersections of users, governance, content, and technology give rise to the four new themes, which all describe elements of translation among the original components. This leads to a theory that the four new translational themes need a great deal of attention if CDS is to live up to its promise. They provide Rich Points for research, for workforce development, and for policy. Figure 2 provides our new model, which includes an oval, symbolizing Translational Interaction, which hovers over the intersections of the four elements. It hovers because it should not obscure the intersections and instead should call attention to them. Insight about each Translational Interaction theme and recommendations follow.

#### Theme 7 Translation for Collaboration

Different stakeholder groups need to share their understandings of CDS. Stakeholders who primarily view CDS as a vehicle for promoting standardization, quality, and safety need to understand that clinicians see it differently and vice versa. This sharing can be done during the processes of decision making about new modules, and of development or modification. Knowledge engineers, even if they are clinicians, should observe and work with users to learn the local workflows and language. Since vendors are collaborators in the CDS process, their perspectives must be understood by organizations.

#### Theme 8 The Meaning of CDS

Because the end users of clinical information systems hold different mental models about CDS from developers, implementers, and organizational decision makers, they cannot always be "on the same page" as these other groups that are responsible for CDS. If users believe the best CDS is that which increases their efficiency and others view CDS as cognitive assistance that sometimes must decrease users' efficiency, there is a large problem. Users are not getting what they need and want and the other groups have difficulty convincing the clinicians that CDS is useful. We urge all stakeholder groups to view CDS broadly. Using our general definition of CDS as "passive and active referential information as well as reminders, alerts, and guidelines [6, p. 524]," we saw excellent uses of CDS in the field, including use of actionable templates in exam rooms in the ambulatory setting. This is a kind of CDS which fits the clinician's mental model as positive, which is being provided by vendors, and which is successful. Our recommendation is that the user view should be considered before CDS is implemented or even developed and that clinicians should be closely involved in any implementation that could possibly decrease efficiency. Also, research is needed to identify different mental models and strategies must be developed for helping to reach a point where a shared mental model exists.

#### Theme 9 Roles of Special, Essential People

The new, emerging roles that center around CDS represent changes in the structure of the health information technology and informatics workforce. Some of the knowledge engineers, pharmacy informaticians, and clinic champions have had no formal informatics training, so we predict that training programs addressing their needs will be needed in the future. Organizations must understand from the beginning that even when they purchase a commercial system, customization will be necessary and knowledge engineers/analysts will be critical. These organizations need to formalize these roles and plan to either create or hire individuals to fill them. By create, we mean provide professional development for them. Finally, individuals with the talent to bridge the gap between the clinical and IT worlds need to consider careers playing these essential roles.

#### Theme 10 Communication, Training, and Support

These are never ending processes as they relate to CDS and they need considerable ongoing resources. Users need to know about current CDS and to be aware of upcoming CDS. Implementers should make sure users are involved from the beginning of the CDS design process, and their feedback should be solicited and taken seriously.

#### Theory and framework

The Multiple Perspectives Framework served to guide us in subject selection and development of questions for interviews and foci for observations, and continuously reminded us of the complex nature of information systems within health care environments. The new construct we have added to the framework, that of Translational Interaction, could be useful for future applied informatics research efforts.

## Limitations

The results of this study, while not generalizable in a quantitative sense, should be transferable to similar contexts. Each recommended practice should be assessed at the local level; sites may need to modify practices depending on their maturity, organizational structure, resources, and information system. The methods were designed to be efficient; results could be different if prolonged periods of time were spent in the field. We did not study sites without EHRs, so our sites are not representative of the majority of hospitals and clinics in the U.S. Because we were funded to only study outpatient sites that belong to the CDS Consortium, and because several of these have locally developed systems with sophisticated CDS, they are not typical of most clinics. On the other hand, these sites provided excellent examples of existing recommended practices.

## Conclusions

In the US, efforts to encourage widespread use of clinical information systems by hospitals and health care providers are likely to succeed only if these systems meet the needs of the major stakeholders. Optimal use and acceptance of clinical decision support is necessary for meaningful use and desired outcomes. For this reason, it is imperative that policy makers, health care administrators, and clinicians reach a mutual shared understanding of CDS and agreement on its goals. A broad view of CDS could include quality and safety aims as well as user workflow assistance, for example. Such aggressive movement will only be possible if the next generation of informatics manpower is available, however. The essential people who will customize, implement, manage and support CDS efforts are key to national efforts and meaningful use of health information technology.

## Competing interests

The authors declare that they have no competing interests.

## Authors' contributions

JSA, DFS, AW, and CM designed and conducted the study, analyzed the data, and wrote the paper. KPG, RHD, JR, JC, AB and MS assisted in the design of the study, gathered and analyzed the data, and reviewed versions of the manuscript. AB also organized the data and contributed significantly to writing the paper. BM, with JSA, obtained funding for the study and reviewed and revised drafts of the manuscript. All authors except RHD, now deceased, have read and approved the final manuscript.

## Pre-publication history

The pre-publication history for this paper can be accessed here:

http://www.biomedcentral.com/1472-6947/12/6/prepub
